# Species Diversity and Molecular Screening of Pyrethroid Resistance Mediated by the Voltage-Gated Sodium Channel in Ixodid Ticks from Puducherry, India

**DOI:** 10.3390/pathogens15060577

**Published:** 2026-05-27

**Authors:** Kaja Hussain Jasmiminal, Elango Ayyanar, Chakravarthi Madda, Vignesh Kumar Ramakrishnan, Hari Kishan Raju Konuganti, Manju Rahi

**Affiliations:** Division of Climate Change, GIS and VBD Stratification/Mapping, National Institute for Vector Control Research (ICMR—NIVCR), Department of Health Research, Indian Council of Medical Research, Puducherry 605006, India; mhaj8254@gmail.com (K.H.J.); elangoar@yahoo.co.in (E.A.); mchakrivrdl@gmail.com (C.M.); victorious.vicky16@gmail.com (V.K.R.); drmanjurahi@gmail.com (M.R.)

**Keywords:** ticks, pyrethroid resistance, Puducherry, species diversity, voltage-gated sodium channel, *kdr* mutations, acaricide resistance, ixodid ticks

## Abstract

Ticks are major ectoparasites of livestock in India, causing substantial economic losses and transmitting a wide range of pathogens. Control strategies rely heavily on synthetic pyrethroid acaricides; however, resistance associated with mutations in the voltage-gated sodium channel (VGSC) gene has been widely reported. Information on tick species diversity and molecular resistance status in Puducherry remains limited. This study investigated tick diversity, host-associated infestation patterns, and the presence of key VGSC knockdown resistance (*kdr*) mutations (C190A and G215T). A cross-sectional survey was conducted across 15 villages in Puducherry, where ticks were collected from cattle, goats, and dogs and identified using standard morpho-taxonomic keys. A total of 3779 ticks representing eight ixodid species were identified, showing clear host-associated infestation patterns. Individual ticks were selected from stratified pools (based on village, host, tick species, and sex) and processed individually for genomic DNA extraction. A fragment of the VGSC gene was amplified by PCR and analysed using Sanger sequencing. Molecular analysis of 62 high-quality VGSC sequences representing all eight species across the surveyed villages revealed no mutations at the investigated loci (C190A and G215T) in the analysed samples. All sequences exhibited the wild-type genotype at these positions. These findings provide baseline molecular information on the screened VGSC loci in ixodid ticks from Puducherry and highlight the importance of continued surveillance, phenotypic validation, and integrated tick management strategies.

## 1. Introduction

Ticks are obligate hematophagous ectoparasites that infest a wide range of mammalian, avian, and reptilian hosts and are of major veterinary and public health importance worldwide [1,2]. In India, they are among the most important ectoparasitic pests affecting cattle, causing substantial economic losses through blood feeding, anaemia, reduced weight gain, decreased milk production, and an overall decline in livestock productivity [3,4]. Beyond their direct parasitic effects, ticks are also efficient vectors of a wide range of pathogens, including viruses, bacteria, rickettsiae, and protozoa, thereby contributing to significant epidemiological and economic burdens [5,6,7].

Importantly, ticks are primary vectors of several economically damaging tick-borne diseases affecting cattle, including bovine babesiosis caused by *Babesia bigemina* and *Babesia bovis*, anaplasmosis caused by *Anaplasma marginale*, and theileriosis caused by *Theileria annulata* and *Theileria orientalis* [8]. These diseases cause significant annual losses in India’s livestock sector through reduced productivity, increased mortality, and higher treatment and management costs [3].

For several decades, chemical acaricides have been the primary strategy for tick control. Among these, synthetic pyrethroids (SPs) such as cypermethrin, deltamethrin, and flumethrin have been widely used due to their effectiveness, rapid knockdown capabilities [9], cost-effectiveness, and more favourable safety profile relative to older organochlorine and organophosphate compounds. However, prolonged and indiscriminate application of these compounds, particularly without veterinary guidance, has exerted substantial selection pressure on tick populations [10]. This has contributed to the emergence of acaricide resistance across multiple geographic regions [11,12]. Species such as *Rhipicephalus (Boophilus) microplus* and *Rhipicephalus australis* are particularly known for their rapid evolutionary response to acaricide exposure, resulting in resistance even under relatively high acaricide concentrations [11,13].

Resistance to multiple acaricide classes has been documented in several tick species worldwide, including *R. (B.) microplus*, *Rhipicephalus (Boophilus) annulatus*, *Rhipicephalus sanguineus*, and *Hyalomma* spp. [14,15,16,17,18,19,20,21,22,23]. These reports highlight the growing challenge of acaricide resistance and the urgent need for continuous monitoring of resistance mechanisms in field tick populations.

One of the most extensively studied mechanisms underlying pyrethroid resistance is target-site insensitivity, commonly known as knockdown resistance (*kdr*). This mechanism arises due to non-synonymous single-nucleotide polymorphisms (SNPs) in the gene encoding the voltage-gated sodium channel (VGSC), the primary neurological target of pyrethroids [24]. Such mutations alter the channel’s conformation, thereby reducing insecticide binding affinity and diminishing their toxic effects. In *Rhipicephalus* species, several VGSC mutations have been implicated in pyrethroid resistance; among them, two mutations located in the domain II S4–5 linker region are considered key molecular markers. These include a C-to-A transversion at nucleotide position 190 (C190A), resulting in a leucine-to-isoleucine substitution (L64I) [25], and a G-to-T transversion at position 215 (G215T), leading to a glycine-to-valine substitution (G72V) [26].

Pyrethroid resistance and associated VGSC mutations, particularly C190A, have been documented in *R. (B.) microplus* populations across several Indian states, including Rajasthan [27], Uttar Pradesh and Maharashtra [19], as well as in reference-resistant tick lines established at IVRI [12]. However, molecular data from South Indian states and Union Territories, including Puducherry, remain limited, representing a significant surveillance gap. The present study addresses this gap by investigating tick species diversity and screening for the occurrence of the VGSC *kdr* mutations C190A and G215T in ixodid ticks collected from Puducherry, India. The findings provide baseline information for future resistance surveillance and tick management in the region.

## 2. Materials and Methods

### 2.1. Study Area and Sample Collection

**Figure 1:**
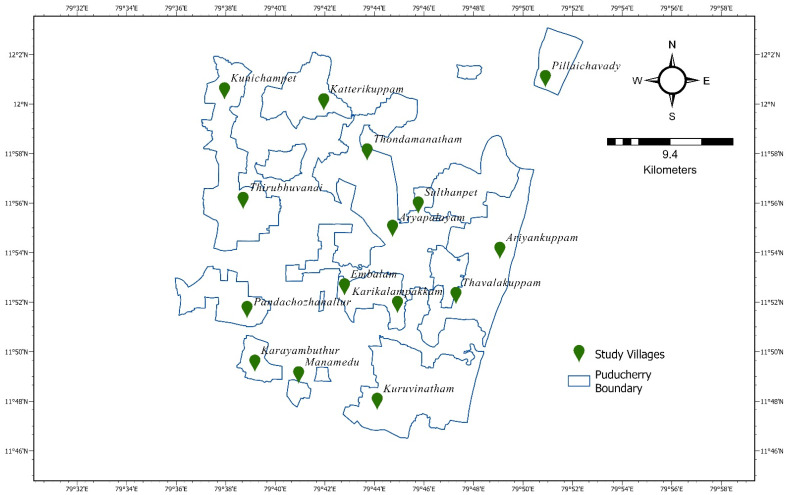
Sampling locations for tick collections screened for resistance study.

A cross-sectional tick survey was conducted in the Union Territory of Puducherry, India. Of the 69 villages within the study region, 15 villages (approximately 20%) were selected for sampling based on livestock census records obtained from the Puducherry Veterinary Department, prioritizing villages with documented populations of cattle, goats, and dogs to ensure adequate host availability for multi-host tick collection (Figure 1).

Tick collection from domestic animals was carried out during the early morning hours of May and June 2025, from 7:00 to 10:00 A.M., when ticks are more readily accessible on hosts. Additionally, information on acaricide usage was collected from livestock owners through structured field interviews conducted at the time of sampling. Data recorded included the type of acaricide used, frequency, and mode of application, and whether treatment practices were guided by a veterinarian or performed independently.

Ticks were manually collected by hand-picking with fine forceps to minimize specimen damage. The collected ticks were preserved in 80% ethanol and transported to the laboratory for subsequent morphological identification and molecular analysis.

### 2.2. Morphological Identification

Under a dissecting microscope, collected ticks were identified to the species level using standard morpho-taxonomic keys [28,29,30]. Ticks were subsequently stratified into pools based on four biological and ecological variables: village, host, tick species, and sex, such that each pool represented a unique combination of these factors (1–25 ticks per pool). A total of 435 pools were formed across all 15 villages to facilitate systematic organisation and downstream processing. Because each pool was internally homogeneous with respect to village, host species, tick species, and sex, any remaining within-pool variation was restricted to individual genomic differences. Random selection of one adult tick per pool therefore enabled systematic and non-redundant representation of each distinct village–host–species–sex stratum within the study area. Individual-level DNA extraction and Sanger sequencing were used to ensure accurate detection of point mutations, as pooled DNA sequencing can generate mixed chromatogram signals that complicate reliable SNP genotyping [31].

For molecular analysis, one adult tick was randomly selected from each pool and processed individually, ensuring broad representation across all stratified sampling groups within the collection. Acaricide application practices, including compound type and treatment intervals, varied across villages and are summarised in Supplementary Table S1.

### 2.3. Calculation of Tick Infestation Indices

Standard parasitological indices were calculated according to established definitions [32,33].

Infestation rate (%) was calculated as:

(1) $$\mathrm{Infestation}\;\mathrm{Rate}\;(\%)=\;\frac{\mathrm{Number}\;\mathrm{of}\;\mathrm{infested}\;\mathrm{animals}}{\mathrm{Total}\;\mathrm{number}\;\mathrm{of}\;\mathrm{animals}\;\mathrm{examined}}\;\times \;100$$

Mean tick abundance was calculated as:

(2) $$\mathrm{Mean}\;\mathrm{Tick}\;\mathrm{Abundance}=\;\frac{\mathrm{Total}\;\mathrm{number}\;\mathrm{of}\;\mathrm{ticks}\;\mathrm{collected}}{\mathrm{Total}\;\mathrm{number}\;\mathrm{of}\;\mathrm{animals}\;\mathrm{examined}}$$

Mean infestation intensity was calculated as:

(3) $$\mathrm{Mean}\;\mathrm{Infestation}\;\mathrm{Intensity}=\;\frac{\mathrm{Total}\;\mathrm{number}\;\mathrm{of}\;\mathrm{ticks}\;\mathrm{collected}}{\mathrm{Number}\;\mathrm{of}\;\mathrm{infested}\;\mathrm{animals}}$$

Confidence intervals (CIs) for infestation rates were computed using the Wilson score method, a recommended approach for proportion data, particularly with small to moderate sample sizes, to account for sampling variability and potential bias associated with non-random host selection [32,34]. All statistical analyses were performed in R software version 4.4.3 [35] using the binom package to calculate binomial confidence intervals.

### 2.4. Genomic DNA Extraction

Genomic DNA was extracted using the DNeasy Blood and Tissue Kit (Qiagen, Hilden, Germany) in accordance with the manufacturer’s instructions. DNA concentration and purity were evaluated using a NanoDrop 2000/2000c spectrophotometer (Thermo Fisher Scientific, Waltham, MA, USA). Samples exhibiting an A260/A280 absorbance ratio of approximately 1.8 were considered to be of sufficient purity and retained for subsequent downstream analyses. Extracted DNA was stored at −20 °C until PCR amplification.

### 2.5. PCR Amplification of VGSC Gene

To screen for pyrethroid resistance-associated mutations, a 167 bp fragment encompassing the domain II S4–S5 linker region of the VGSC gene was amplified by Polymerase Chain Reaction (PCR). Amplification was carried out using the following primers RmNaDomainIIF1 (forward: 5′-TACGTGTGTTCAAGCTAGCCAA-3′) and RmNaDomainIIR1 (reverse: 5′-ACTTTCTTCGTAGTTCTTGCCAA-3′), as previously described [31].

The PCR was performed in a 25 µL reaction mixture comprising 12.5 µL of 2X Taq DNA polymerase master mix (AMPLIQON), 1.5 µL of each primer (10 pmol/µL), 2.0 µL of template DNA, and 9 µL of nuclease-free water. The cycling conditions included an initial denaturation step at 95 °C for 10 min, followed by 40 amplification cycles consisting of denaturation at 94 °C for 60 s, annealing at 55 °C for 30 s, and extension at 72 °C for 30 s to ensure complete strand synthesis.

Amplified products were subjected to electrophoresis on an ethidium bromide-stained 1.5% agarose gel, then visualized and recorded using a gel documentation system.

### 2.6. Sequencing and Data Analysis

PCR-positive amplicons from individually processed ticks were purified using the NucleoSpin Gel and PCR Clean-up Kit (Macherey-Nagel, Düren, Germany) according to the manufacturer’s instructions. Purified 167 bp amplicons were then subjected to bidirectional Sanger sequencing using the BigDye Terminator v3.1 Cycle Sequencing Kit (Applied Biosystems, Thermo Fisher Scientific, Waltham, MA, USA).

Samples were retained for mutation analysis only when high-quality forward and reverse electropherograms were obtained for both strands. This quality-filtering process yielded 62 high-quality sequences, selected to represent unique village–species combinations across the 15 surveyed villages (Supplementary Table S2).

Forward and reverse sequences were assembled into consensus sequences and aligned using the ClustalW algorithm implemented in MEGA version 11 (Molecular Evolutionary Genetics Analysis, Pennsylvania State University, University Park, PA, USA). The consensus sequences were compared with reference sequences from the National Center for Biotechnology Information (NCBI) GenBank database to confirm the identity of the amplified VGSC gene fragment and to screen for SNPs at positions corresponding to the C190A and G215T mutations.

Phylogenetic analysis was performed using the Maximum Likelihood method based on the Tamura–Nei substitution model in MEGA version 11 to assess the genetic relationships between the study sequences and previously reported resistant and susceptible VGSC sequences. The robustness of the inferred tree topology was evaluated using 1000 bootstrap replicates.

## 3. Results

### 3.1. Tick Collection and Host Infestation Rate

In total, 3779 ticks were collected from 552 domestic animals, comprising 413 cattle, 124 goats, and 15 dogs. The overall infestation rate was 36.59% (95% CI: 32.68–40.69). Among the examined hosts, dogs had the highest infestation rate at 60.00% (95% CI: 35.75–80.18); however, this estimate should be interpreted with caution given the small number of dogs examined (n = 15), reflected by the wide confidence interval. This was followed by cattle at 40.92% (95% CI: 36.28–45.72), while goats had the lowest infestation rate at 19.35% (95% CI: 13.37–27.19) (Table 1).

### 3.2. Species Diversity and Proportional Representation

The survey identified a total of eight tick species, including two *Haemaphysalis* species, one *Hyalomma* species, and five *Rhipicephalus* species. *Hyalomma kumari* was recorded as a rare incidental species, represented by only four specimens from a single village (Katterikuppam), and should be considered an uncommon and geographically limited component of the local tick fauna. The most abundant species among these was *Haemaphysalis bispinosa,* accounting for 40.86% of the total samples collected, followed by *R. (B.) annulatus* (38.82%) and *Haemaphysalis intermedia* (16.88%) (Table 2).

### 3.3. Mean Abundance and Mean Infestation Intensity

The mean tick abundance was found to be 6.85 ticks per host, with *H. bispinosa* being the most abundant species (2.80 ticks per host), followed by *R. (B.) annulatus* (2.66 ticks per host) and *H. intermedia* (1.16 ticks per host). The overall mean infestation intensity was 18.71 ticks per infested host; the highest mean intensities were found in *H. bispinosa* and *R. (B.) annulatus* (7.64 and 7.26 ticks per infested host, respectively) (Table 3).

At the host level, cattle had the highest mean tick abundance (8.42 ticks per host) and infestation intensity (20.57 ticks per infested host), with infestations dominated by *H. bispinosa* and *R. (B.) annulatus*. Tick infestations in goats were less intense, with a mean tick abundance of 2.08 and an infestation intensity of 10.75 ticks per infested host, with *H. intermedia* dominating the infestation. Dogs had a mean tick abundance of 3.00 ticks per host and a mean infestation intensity of 5.00 ticks per infested host, with *R. sanguineus* predominating (Supplementary Table S3).

### 3.4. Acaricide Usage Patterns

Acaricide usage patterns across the 15 surveyed villages are summarised in Supplementary Table S1. Pyrethroid-based acaricides, particularly flumethrin and permethrin, along with amitraz, were the most frequently reported compounds. Application intervals ranged from 7 to 25 days; amitraz was applied at shorter intervals (7–10 days) in several villages, while injectable macrocyclic lactones (ivermectin and doramectin) were used at longer intervals of 3–4 weeks.

Information from field interviews indicated that acaricide application in all surveyed villages was conducted under veterinary supervision. Livestock owners reported receiving advice on product selection and application schedules from government veterinary practitioners during periodic visits. Multiple acaricide classes including pyrethroids, amitraz, and macrocyclic lactones, were used across villages, with variation in compound type and application frequency.

### 3.5. Molecular Screening for VGSC Mutations

**Figure 2:**
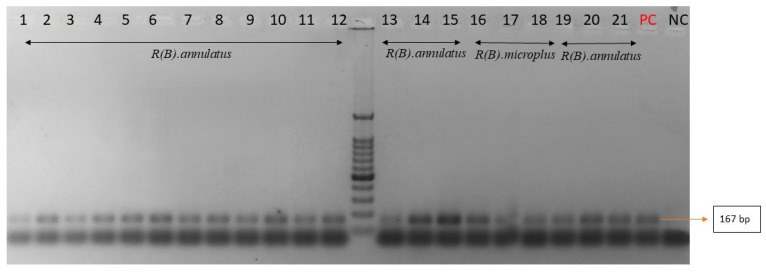
Agarose gel electrophoresis showing successful PCR amplification of the expected 167 bp VGSC gene fragment from representative *R. (B.) annulatus* and *R. (B.) microplus* samples. The presence of the expected amplicon confirms successful amplification of the target VGSC domain II S4–S5 linker region in cattle-associated *Rhipicephalus* species. PC: positive control; NC: negative control.

**Figure 3:**
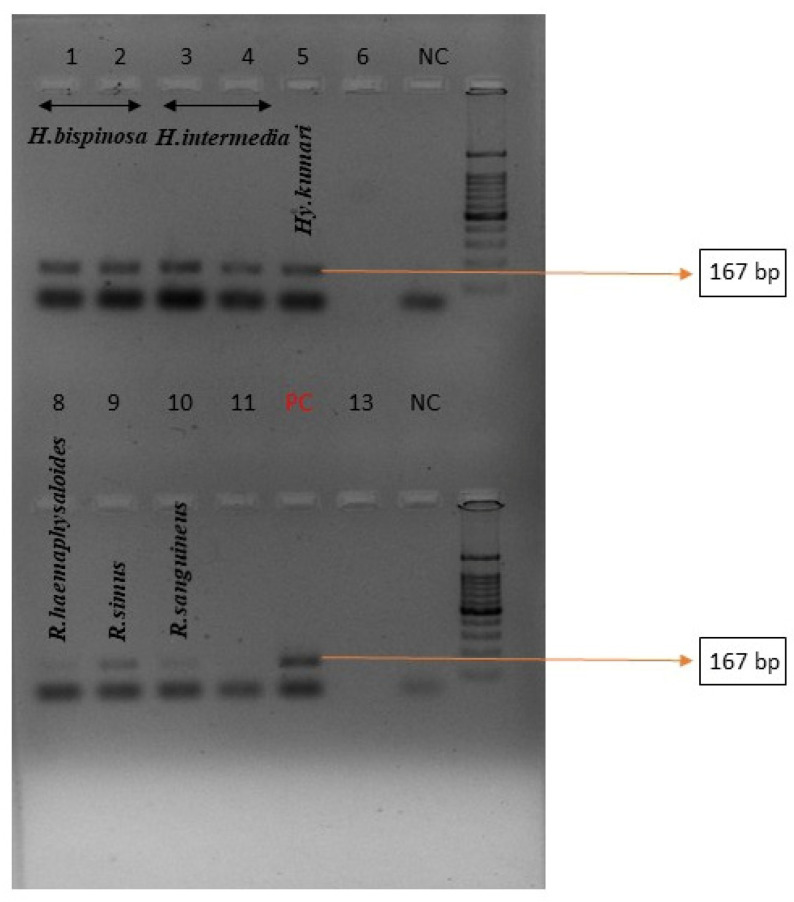
Agarose gel electrophoresis showing successful PCR amplification of the expected 167 bp VGSC gene fragment from representative *H. bispinosa*, *H. intermedia*, *Hy. kumari*, *R. haemaphysaloides*, *R. simus*, and *R. sanguineus* samples. Successful amplification across multiple ixodid genera demonstrates the cross-species applicability of the VGSC primers targeting the domain II S4–S5 linker region used for *kdr* mutation screening. PC: positive control; NC: negative control.

**Figure 4:**
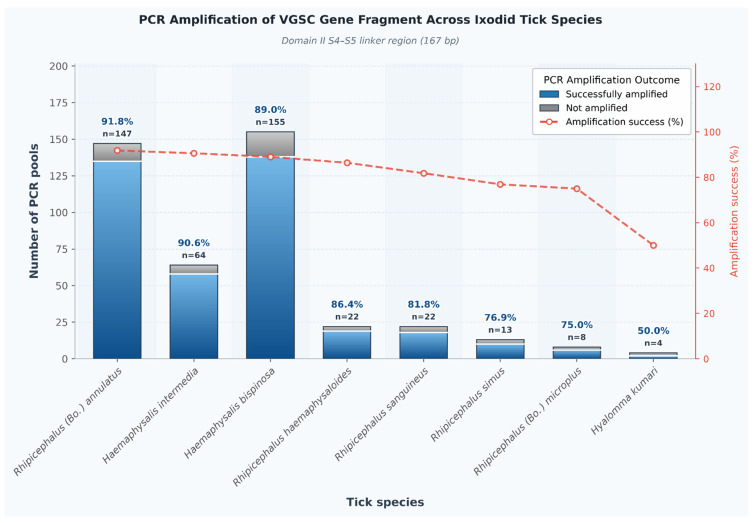
PCR amplification success of the voltage-gated sodium channel (VGSC) gene fragment across eight ixodid tick species. Bars represent the number of PCR-positive and PCR-negative pools screened for each species, while the dashed line indicates amplification success percentage. Successful amplification across phylogenetically diverse ixodid taxa demonstrates the applicability of the VGSC primers for multi-species molecular resistance surveillance. Sample sizes for each species are indicated within the bars.

From the collected samples, genomic DNA was extracted, with concentrations ranging from 35.3 ng/µL to 90.8 ng/µL and A260/A280 purity ratios from 1.75 to 1.9. The PCR assay using RmNaDomainIIF1/R1 primers produced the expected 167 bp VGSC amplicon across all eight tick species (Figure 2 and Figure 3), confirming cross-species amplification capacity across phylogenetically diverse ixodid genera. Amplification success at the individual sample level varied across species, ranging from 50.0% in *Hy. kumari* (n = 4 pools) to 91.8% in *R. (B.) annulatus* (Figure 4). The comparatively lower success rates observed in *Haemaphysalis* and *Hyalomma* species likely reflect sequence divergence in the primer-binding regions flanking the target locus, as these primers were originally designed for *R. (B.) microplus* [31].

A total of 62 samples yielded high-quality bidirectional VGSC sequences and were included in mutation analysis. These sequences were selected to represent unique village–species combinations across the 15 surveyed villages. The analysed samples comprised eight tick species: *R. (B.) annulatus* (n = 15), *H. bispinosa* (n = 14), *H. intermedia* (n = 13), *R. sanguineus* (n = 6), *Rhipicephalus simus* (n = 6), *Rhipicephalus haemaphysaloides* (n = 6), *R. (B.) microplus* (n = 1), and *Hy. kumari* (n = 1). The number of analysed sequences therefore varied across species, with higher representation for *R. (B.) annulatus*, *H. bispinosa*, and *H. intermedia*, and single representative sequences for *R. (B.) microplus* and *Hy. kumari*.

**Figure 5:**
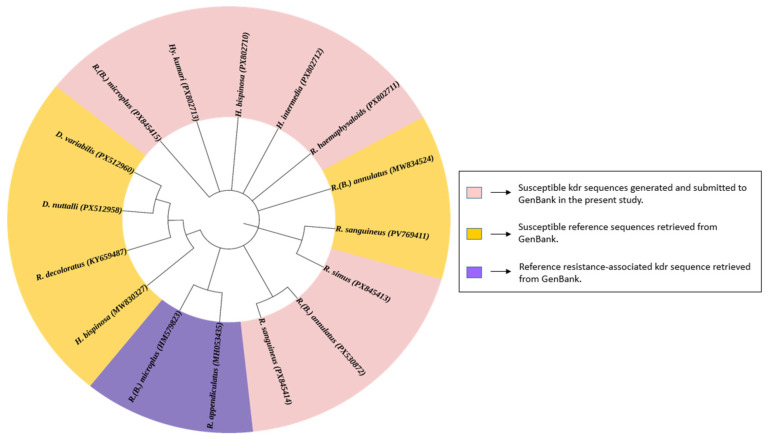
Maximum-likelihood phylogenetic analysis of partial VGSC sequences obtained from ixodid ticks in Puducherry together with reference susceptible and resistant sequences. Study sequences clustered with susceptible reference sequences within their respective genera, while resistant reference sequences remained distinct, consistent with the absence of screened VGSC resistance-associated mutations in the analysed dataset.

The highest-quality sequences were submitted to the NCBI database (Table 4). Multiple sequence alignment with reference sequences confirmed that the amplified fragments corresponded to the VGSC gene. Phylogenetic analysis showed genus-level clustering of the study sequences with reference sequences (Figure 5).

**Figure 6:**
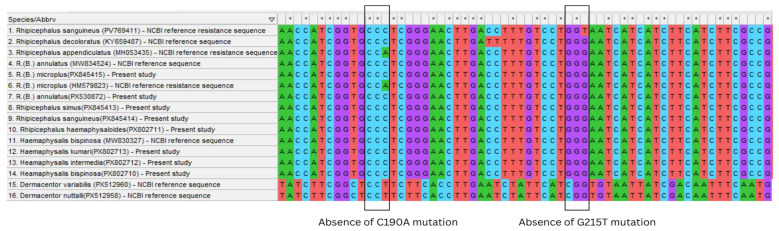
Multiple sequence alignment of partial VGSC gene sequences showing conservation of the C190 and G215 loci across all analysed ixodid tick species. No nucleotide substitutions corresponding to the C190A (L64I) or G215T (G72V) *kdr*-associated mutations were detected in the analysed samples. Nucleotide positions are colour-coded according to base identity (ClustalW scheme), and asterisks (*) indicate positions conserved across all aligned sequences. The boxed regions indicate the positions corresponding to the C190 and G215 loci.

No mutations corresponding to the C190A (L64I) and G215T (G72V) loci were detected in any of the 62 analysed sequences. All sequences exhibited the wild-type cytosine (C) at position 190 and guanine (G) at position 215, as confirmed by bidirectional Sanger sequencing and multiple sequence alignment (Figure 6).

## 4. Discussion

### 4.1. Tick Species Diversity and Host Associations

This study provides the first molecular screening of VGSC-associated pyrethroid resistance across multiple ixodid tick species in the Union Territory of Puducherry, India. Eight ixodid tick species were identified on cattle, goats, and dogs, revealing distinct host-associated infestation patterns. These findings are consistent with an earlier regional survey from Puducherry [36], which similarly documented eight ixodid species infesting cattle and other domestic animals. The substantial overlap in species composition, including *R. sanguineus*, *R. (B.) annulatus*, *R. (B.) microplus*, *R. haemaphysaloides*, *R. simus*, *H. bispinosa*, and *H. intermedia*, suggests relative consistency in regional tick fauna composition despite differences in sampling period and host composition.

Cattle infestations were dominated by *H. bispinosa* and *R. (B.) annulatus*, which together accounted for the majority of the total tick burden. Similar host–tick associations have been reported from other regions of southern India, where bovine hosts frequently harbour high burdens of *R. (B.) annulatus* and *Haemaphysalis* species [36,37]. These patterns likely reflect the ecological adaptation of these tick species to large ruminant hosts and livestock-associated environments. *H. intermedia* predominated in goat infestations [38], whereas dogs were parasitized predominantly by *R. sanguineus*, consistent with its recognised host preference and widespread global distribution [39]. Collectively, these recurring patterns indicate that host ecology, behaviour, and management practices strongly influence local tick community composition, highlighting the importance of host-specific rather than uniform tick control strategies.

Variation in species richness across surveyed villages (Supplementary Table S2) appeared to reflect differences in local ecological and management conditions. Villages with higher species richness were predominantly rural and characterised by greater livestock density, mixed host availability, and proximity to vegetated or semi-natural habitats, all of which provide favourable microclimatic conditions for tick survival and development. In contrast, lower species richness in urban or peri-urban settings may be associated with reduced host diversity, lower vegetation cover, and increased anthropogenic disturbance, factors known to constrain tick persistence and habitat suitability. Comparable patterns of greater tick diversity in rural livestock systems relative to urbanised settings have been reported in India and other regions [36,37]. These observations underscore the role of environmental heterogeneity and host availability in shaping local tick assemblages. Villages with higher species richness often exhibited more diverse host composition and varied acaricide usage patterns (Supplementary Tables S1 and S2), potentially contributing to heterogeneous selection environments across tick species and sites.

The host-associated infestation patterns observed in the present study also have implications for understanding potential resistance selection dynamics. Cattle exhibited the highest mean infestation intensity and harboured the most abundant tick species, particularly *H. bispinosa* and *R. (B.) annulatus*, while pyrethroid-based acaricides were reported to be used predominantly in cattle management systems within the study area (Supplementary Table S1). Consequently, cattle-associated tick populations are likely to be exposed more frequently to acaricide treatment than ticks associated with other hosts. The absence of C190A and G215T mutations in the more extensively sampled cattle-associated species therefore provides important baseline molecular information for future resistance surveillance. In contrast, *R. sanguineus*, which was detected primarily on dogs and represented by fewer analysed sequences, has previously been reported to carry VGSC mutations in Brazil [23], highlighting the importance of continued monitoring across different host-associated tick species.

### 4.2. Absence of Detectable kdr Mutations in Tick Populations from Puducherry

Despite high infestation intensities in the dominant cattle-associated species (*H. bispinosa*: 7.64 ticks per infested host; *R. (B.) annulatus*: 7.26 ticks per infested host; Table 2) and regular acaricide application under veterinary supervision across all villages (Supplementary Table S1), molecular screening revealed no evidence of the C190A or G215T mutations in any of the 62 analysed sequences.

The strength of inference, however, varies with sample size. For the three most extensively sampled species: *R. (B.) annulatus* (n = 15), *H. bispinosa* (n = 14), and *H. intermedia* (n = 13), the absence of mutations across multiple individuals from different villages provides stronger support for the presence of wild-type genotypes in the analysed subset. For *R. sanguineus*, *R. simus*, and *R. haemaphysaloides* (n = 6 each), the absence of mutations does not exclude the presence of low-frequency resistance alleles. For *R. (B.) microplus* and *Hy. kumari* (n = 1 each), sequencing confirms the genotype of a single individual only, and no population-level inference can be made.

These findings should therefore be interpreted with caution. The analysis was limited to a subset of samples and to two VGSC loci; consequently, low-frequency variants or resistance mediated through alternative mechanisms, including metabolic detoxification pathways, cannot be excluded [40]. Importantly, the absence of detected target-site mutations does not necessarily indicate the absence of selection pressure, but may reflect differences in resistance pathways or local selection dynamics.

### 4.3. Indian and Global Perspectives on Acaricide Resistance

The absence of VGSC-associated *kdr* mutations in the present study contrasts with reports from several regions of India and other parts of the world, where moderate to high frequencies of these mutations have been documented, particularly in *R. (B.) microplus* populations [12,13,27,31]. In India, the C190A mutation has been reported in *R. (B.) microplus* populations from Rajasthan [27] and Uttar Pradesh [19], while experimentally selected resistant lines maintained at IVRI have confirmed the presence of both C190A and G215T mutations under selection pressure [12]. Resistance-associated mutations have also been identified in *Hyalomma anatolicum* from Gujarat [20], indicating that VGSC-mediated resistance is not restricted to a single genus within the Indian subcontinent. Globally, *kdr*-type mutations have been documented in tick populations across Australia, Latin America, the United States, Mexico [13,31], and Southeast Asia [22], and have recently been reported in *R. sanguineus* from Brazil [23].

Despite this broader pattern, knowledge of VGSC-mediated resistance remains uneven across tick genera. While *Rhipicephalus (Boophilus)* species have been extensively investigated, molecular evidence for resistance in *Haemaphysalis* species remains limited, despite their substantial representation in the present study. The detection of wild-type genotypes at the screened loci in *H. bispinosa* and *H. intermedia* therefore provides preliminary molecular baseline information for these species in Puducherry. Whether differences in life cycle, host preference, and acaricide exposure influence resistance development across genera remains unclear. Notably, the domain II S4–S5 linker region targeted in the present study is structurally conserved across ixodid genera, as confirmed by successful cross-species amplification, suggesting that if equivalent selection pressure were applied, analogous target-site mutations could theoretically arise in *Haemaphysalis* species through the same molecular pathway reported in *Rhipicephalus* [24,25].

Successful amplification of the VGSC domain II S4–S5 region across phylogenetically diverse ixodid taxa, including *Haemaphysalis*, *Hyalomma*, and multiple *Rhipicephalus* species, indicates conservation of this target region across genera. This suggests that molecular markers commonly applied in *Rhipicephalus*-based resistance studies may also be useful for broader multi-species surveillance programmes in regions where non-*Rhipicephalus* ticks are ecologically important but remain understudied at the molecular level. Previous surveys from neighbouring regions of South India, including Tamil Nadu [37,38], have documented tick fauna composition broadly comparable to that observed in Puducherry; however, molecular data on VGSC-associated resistance from these regions remain limited.

Field observations from the present study (Supplementary Table S1) indicate that multiple acaricide classes are used across villages under veterinary guidance. Variation in compound usage and application intervals may influence the nature and intensity of selection pressure acting on local tick populations. While such practices are consistent with approaches associated with reduced selection on individual resistance targets in other livestock systems [10], their relationship to the absence of detectable VGSC mutations cannot be established directly from the present data.

The absence of detectable VGSC mutations in this study may reflect several non-exclusive factors, including maintenance of susceptibility under current management practices, the presence of resistance alleles at frequencies below the detection threshold of the sampled population [31], involvement of alternative resistance mechanisms such as metabolic detoxification pathways [11], or limited introduction of resistant alleles due to geographic or management related factors [10]. Further studies incorporating larger sample sizes, phenotypic bioassays, and investigation of metabolic resistance pathways are required to clarify these possibilities.

### 4.4. Ecological and Management Implications

The absence of detected VGSC target-site mutations in the analysed subset, together with the observed tick species diversity, provides a useful baseline for resistance monitoring in the study region. Early stage surveillance prior to the establishment of resistance can support the timely implementation of evidence-based management strategies. Approaches such as rotation of acaricide classes, adherence to recommended dosing practices, and integration of non-chemical control measures may help delay the emergence of resistance in local tick populations.

Phylogenetic analysis of the VGSC gene fragment showed genus-level clustering of the study sequences with reference sequences, indicating consistency of the amplified fragment across taxa. The grouping of study sequences with reference sequences lacking known resistance-associated mutations is consistent with the absence of the screened VGSC mutations in the analysed samples. However, these findings are based on a partial gene fragment and a limited number of sequences, and therefore do not exclude the presence or spread of resistance-associated alleles within the broader tick population.

### 4.5. Limitations and Future Directions

Several limitations warrant consideration. First, the number of sequences analysed per species was limited, particularly for some taxa, which reduces the ability to detect low-frequency resistance alleles and constrains population-level inference. For example, a resistance allele present at 5% population frequency would be detected with only approximately 26% probability in a sample of n = 6 (*P* = 1 − 0.95^6^), and with less than 10% probability in a sample of n = 1. Larger sample sizes will therefore be necessary in future studies to improve estimation of resistance allele frequencies within tick populations.

Second, molecular screening was restricted to two well-characterised SNPs (C190A and G215T) in the domain II S4–S5 linker region of the VGSC gene. Other VGSC mutations associated with pyrethroid resistance in ticks globally, including those identified in domain I and domain III, were not screened and therefore cannot be excluded [31].

Third, metabolic resistance mechanisms, including those mediated by cytochrome P450 monooxygenases, esterases, and glutathione S-transferases, were not investigated; their contribution to any field-level pyrethroid tolerance in these populations therefore remains unknown.

Fourth, no phenotypic bioassays, such as the larval packet test (LPT) or larval tarsal test (LTT), were conducted [40]. The absence of detected mutations at the screened VGSC loci cannot be equated with confirmed pyrethroid susceptibility of tick populations, and phenotypic assays remain an essential complement to molecular screening in resistance monitoring.

Fifth, the one-tick-per-pool design, while enabling broad coverage across sampling strata, does not permit estimation of allele frequencies within pools or within village-level populations; the present data therefore reflect only the genotype of individually tested specimens. Finally, the cross-sectional design of this survey precludes assessment of temporal trends in resistance allele emergence. Longitudinal monitoring, expanded geographic sampling, inclusion of phenotypic bioassays, and investigation of metabolic resistance pathways will be important for a more comprehensive evaluation of acaricide resistance dynamics in tick populations of Puducherry.

## 5. Conclusions

This study provides baseline evidence that the two major VGSC *kdr* mutations, C190A (L64I) and G215T (G72V), were not detected in 62 individually screened ixodid ticks representing eight species from 15 villages in Puducherry, India. The absence of these target-site substitutions in the analysed specimens, together with the observed acaricide usage patterns in the region, indicates that the screened VGSC resistance-associated mutations were not detected in the sampled individuals. However, these findings should be interpreted cautiously and do not establish phenotypic susceptibility; confirmation through bioassays is required, and the potential contribution of alternative resistance mechanisms cannot be excluded.

The documented eight-species diversity and clear host-associated infestation patterns provide an ecological framework for developing targeted tick management strategies. Given the increasing global emergence of acaricide resistance, particularly in systems with sustained use of single chemical classes, continued surveillance remains important even in regions where resistance has not been detected at the screened loci. The present findings establish an initial reference point for future monitoring of resistance-associated alleles in Puducherry. Integration of molecular surveillance with phenotypic bioassays and evidence-based tick control practices will be important for maintaining the long-term effectiveness of current management strategies in the region.

## Figures and Tables

**Table 1:** Infestation rate of ixodid ticks among domestic animals in Puducherry, India, with Wilson 95% confidence intervals.

Host	No. of Host Examined	No. of Host Infested	Infestation Rate (%)	Lower 95% CI	Upper 95% CI
Cattle	413	169	40.92	36.28	45.72
Dog	15	9	60.00	35.75	80.18
Goat	124	24	19.35	13.37	27.19
**Total**	**552**	**202**	**36.59**	**32.68**	**40.69**

**Table 2:** Host-wise species composition and proportional representation of ixodid ticks collected from domestic animals in Puducherry, India.

Species	Cattle		Goat		Dog		Total	
	No. of Ticks	0%	No. of Ticks	%	No. of Ticks	%	No. of Ticks	%
*Haemaphysalis bispinosa*	1505	43.30	39	15.12	0	0.00	1544	40.86
*Haemaphysalis intermedia*	424	12.20	208	80.62	6	13.33	638	16.88
*Hyalomma kumari*	4	0.12	0	0.00	0	0.00	4	0.11
*Rhipicephalus (Bo.) annulatus*	1467	42.20	0	0.00	0	0.00	1467	38.82
*Rhipicephalus (Bo.) microplus*	13	0.37	0	0.00	0	0.00	13	0.34
*Rhipicephalus sanguineus*	15	0.43	3	1.16	25	55.56	43	1.14
*Rhipicephalus simus*	8	0.23	4	1.55	14	31.11	26	0.69
*Rhipicephalus haemaphysaloides*	40	1.15	4	1.55	0	0.00	44	1.16
**Grand Total**	**3476**	**91.98**	**258**	**6.83**	**45**	**1.19**	**3779**	**100.00**

**Table 3:** Mean abundance and mean infestation intensity of ixodid tick species collected from domestic animals in Puducherry, India.

Species	Mean Abundance	Mean Infestation Intensity
*Haemaphysalis bispinosa*	2.80	7.64
*Haemaphysalis intermedia*	1.16	3.16
*Hyalomma kumari*	0.01	0.02
*Rhipicephalus (Bo.) annulatus*	2.66	7.26
*Rhipicephalus (Bo.) microplus*	0.02	0.06
*Rhipicephalus sanguineus*	0.08	0.21
*Rhipicephalus simus*	0.05	0.13
*Rhipicephalus haemaphysaloides*	0.08	0.22
**Grand Total**	**6.85**	**18.71**

**Table 4:** Details of VGSC gene sequences generated from different ixodid tick species and submitted to the NCBI database.

Species Name	Gene Name	Accession Number	Base Pair
*Haemaphysalis bispinosa*	Domain II of Voltage gated sodium channel	PX802710	148 bp
*Haemaphysalis intermedia*	Domain II of Voltage gated sodium channel	PX802712	167 bp
*Hyalomma kumari*	Domain II of Voltage gated sodium channel	PX802713	166 bp
*Rhipicephalus (Bo.) annulatus*	Domain II of Voltage gated sodium channel	PX530872	163 bp
*Rhipicephalus (Bo.) microplus*	Domain II of Voltage gated sodium channel	PX845415	167 bp
*Rhipicephalus sanguineus*	Domain II of Voltage gated sodium channel	PX845414	154 bp
*Rhipicephalus simus*	Domain II of Voltage gated sodium channel	PX845413	148 bp
*Rhipicephalus haemaphysaloides*	Domain II of Voltage gated sodium channel	PX802711	140 bp

## Data Availability

Data will be available upon reasonable request from the Corresponding Author.

## Supplementary Materials

The following supporting information can be downloaded at https://www.mdpi.com/article/10.3390/pathogens15060577/s1. Table S1: Type and Frequency of Acaricide application across surveyed villages; Table S2: Tick Species richness in surveyed villages; Table S3: Host-wise species composition, mean abundance, and mean infestation intensity of ixodid ticks collected from domestic animals in Puducherry, India.

## Abbreviations

The following abbreviations are used in this manuscript:

PCR	Polymerase Chain Reaction
VGSC	Voltage-gated Sodium Channel
*kdr*	Knock Down Resistance
CI	Confidence Interval
SNP	Single-nucleotide polymorphisms
NCBI	National Center for Biotechnology Information
IVRI	Indian Veterinary Research Institute
LPT	larval packet test
LTT	larval tarsal test
SPs	synthetic pyrethroids
