# Supplementary material for: Species Diversity and Molecular Screening of Pyrethroid Resistance Mediated by the Voltage-Gated Sodium Channel in Ixodid Ticks from Puducherry, India
Source: Pathogens. 2026 May 27;15(6):577. doi: 10.3390/pathogens15060577 (PMC13305014; doi:10.3390/pathogens15060577)
Supplement: Supplementary file 1 [file pathogens-15-00577-s001.zip › pathogens-4295932-supplementary.pdf]

Supplementary Table S1. Type and Frequency of Acaricide application across surveyed villages.

Village Name	Type of Acaricide used	Frequency of Acaricide application
Katterikuppam	Flumethrin	21 days
	Amitraz	7 days
	Cypermethrin	10 days
Thondamanatham	Flumethrin	21 days
	Amitraz	7 days
	Cypermethrin	10 days
	Ivermectin	4 weeks
	Deltamethrin	14 days
Kunichanpet	Permethrin	14 days
	Amitraz	10 days
Thirubhuvanai	Flumethrin	14 days
	Permethrin	12 days
	Amitraz	15 days
Pandachozhanallur	Flumethrin	14 days
	Amitraz	7 days
	Permethrin	7 days
Manamedu	Amitraz	7 days
	Flumethrin	14 days
	Permethrin	7 days
	Doramectin	3 weeks
Karayambuthur	Amitraz	10 days
	Permethrin	14 days
	Flumethrin	21 days
Sulthanpet	Amitraz	10 days
	Permethrin	15 days
Embalam	Flumethrin	15 days

	Amitraz	7 days
Ariyankuppam	Amitraz	10 days
	Flumethrin	20 days
Kuruvinatham	Amitraz	10 days
	Permethrin	15 days
	Flumethrin	25 days
Thavalakuppam	Permethrin	14 days
	Amitraz	12 days
	Deltamethrin	14 days
Karikalampakkam	Amitraz	7 days
	Flumethrin	20 days
Aryapalayam	Permethrin	14 days
	Flumethrin	25 days
Pillaichavady	Amitraz	8 days
	Flumethrin	15 days

Supplementary Table S2. Tick Species richness in surveyed villages.

Village name	Host	Species richness	Species
Katterikuppam	Cattle<br>Goat	5	*H. bispinosa*<br>*H. intermedia*<br>*R. (B.) annulatus*<br>*R. haemaphysaloides*<br>*Hy. kumari*
Thondamanatham	Cattle<br>Goat	4	*H. bispinosa*<br>*H. intermedia*<br>*R. (B.) annulatus*<br>*R. sanguineus*
Kunichanpet	Cattle<br>Goat	4	*H. bispinosa*<br>*H. intermedia*<br>*R. (B.) annulatus*<br>*R. sanguineus*
Thirubhuvanai	Cattle<br>Goat	4	*H. bispinosa*<br>*H. intermedia*<br>*R. (B.) annulatus*<br>*R. haemaphysaloides*
Pandachozhanallur	Cattle<br>Goat	4	*H. bispinosa*<br>*H. intermedia*<br>*R. (B.) annulatus*<br>*R. simus*
Manamedu	Cattle<br>Dog	4	*H. intermedia*<br>*R. (B.) annulatus*<br>*R. haemaphysaloides*<br>*R. simus*

Karayambuthur	Cattle<br>Goat<br>Dog	5	*H. bispinosa*<br>*H. intermedia*<br>*R. (B.) annulatus*<br>*R. sanguineus*<br>*R. simus*
Sulthanpet	Cattle<br>Goat	3	*H. bispinosa*<br>*H. intermedia*<br>*R. (B.) annulatus*
Embalam	Cattle<br>Dog	4	*H. bispinosa*<br>*R. (B.) annulatus*<br>*R. haemaphysaloides*<br>*R. simus*
Ariyankuppam	Cattle	3	*H. bispinosa*<br>*R (B).annulatus*<br>*R. simus*
Kuruvinatham	Cattle<br>Goat	4	*H. bispinosa*<br>*H. intermedia*<br>*R (B).annulatus*<br>*R. haemaphysaloides*
Thavalakuppam	Cattle	5	*H. bispinosa*<br>*H. intermedia*<br>*R. (B.) annulatus*<br>*R. sanguineus*<br>*R. simus*
Karikalampakkam	Cattle	4	*H. bispinosa*<br>*H. intermedia*<br>*R. (B.) annulatus*<br>*R. haemaphysaloides*
Aryapalayam	Cattle	5	*H. bispinosa*<br>*H. intermedia*<br>*R. (B.) microplus*<br>*R. (B.) annulatus*<br>*R. sanguineus*

Pillaichavady	Cattle<br>Goat	4	*H. bispinosa*<br>*H. intermedia*<br>*R. (B.) annulatus*<br>*R. sanguineus*

Supplementary Table S3. Host-wise species composition, mean abundance, and mean infestation intensity of ixodid ticks collected from domestic animals in Puducherry, India.

Species Name	Cattle				Goat				Dog							
	No. of Ticks	%	Mean Abundance	Mean Intensity	No. of Ticks	%	Mean Abundance	Mean Intensity	No. of Ticks	%	Mean Abundance	Mean Intensity	Total No. of Ticks	Total %	Total Mean Abundance	Total Mean Intensity
Haemaphysalis bispinosa	1505	43.30	3.64	8.91	39	15.12	0.31	1.63	0	0.00	0.0	0.00	1544	40.86	2.80	7.64
Haemaphysalis intermedia	424	12.20	1.03	2.51	208	80.62	1.68	8.67	6	13.33	0.40	0.67	638	16.88	1.16	3.16
Hyalomma kumari	4	0.12	0.01	0.02	0	0.00	0.00	0.00	0	0.00	0.00	0.00	4	0.11	0.01	0.02
Rhipicephalus (Bo.) annulatus	1467	42.20	3.55	8.68	0	0.00	0.00	0.00	0	0.00	0.00	0.00	1467	38.82	2.66	7.26
Rhipicephalus (Bo.) microplus	13	0.37	0.03	0.08	0	0.00	0.00	0.00	0	0.00	0.00	0.00	13	0.34	0.02	0.06
Rhipicephalus sanguineus	15	0.43	0.04	0.09	3	1.16	0.02	0.13	25	55.56	1.67	2.78	43	1.14	0.08	0.21

Rhipicephalus simus	8	0.23	0.02	0.05	4	1.55	0.03	0.17	14	31.11	0.93	1.56	26	0.69	0.05	0.13
Rhipicephalus haemaphysaloides	40	1.15	0.10	0.24	4	1.55	0.03	0.17	0	0.00	0.00	0.00	44	1.16	0.08	0.22
**Grand Total**	**3476**	**100.00**	**8.42**	**20.57**	**258**	**100.00**	**2.08**	**10.75**	**45**	**100.00**	**3.00**	**5.00**	**3779**	**100.00**	**6.85**	**18.71**
